# Auto-Correction of 3D-Orientation of IMUs on Electric Bicycles †

**DOI:** 10.3390/s20030589

**Published:** 2020-01-21

**Authors:** Jan Schnee, Jürgen Stegmaier, Tobias Lipowsky, Pu Li

**Affiliations:** 1 Robert Bosch GmbH, 70839 Gerlingen-Schillerhoehe, Germany; juergen.stegmaier2@de.bosch.com (J.S.); tobias.lipowsky@de.bosch.com (T.L.); 2 Institute for Automation and System Engineering, Ilmenau University of Technology, 98693 Ilmenau, Germany; pu.li@tu-ilmenau.de

**Keywords:** IMU, mounting estimation, electric bicycle, MEMS, orientation, calibration

## Abstract

The application of inertial measurement units (IMU) in electronically power-assisted cycles (EPACs) has become increasingly important for improving their functionalities. One central issue of such an application is to calibrate the orientation of the IMU on the EPAC. The approach presented in this paper utilizes common bicycling motions to calibrate the 2D- and 3D-mounting orientation of a micro-electro-mechanical system (MEMS) IMU on an electric bicycle. The method is independent of sensor biases and requires only a very low computation expense and, thus, the estimation can be realized in real-time. In addition, the acceleration biases are estimated using a barometric pressure sensor. The experimental results show high accuracy of the calibrated orientation and estimated sensor biases.

## 1. Introduction

Applications of inertial measurement units (IMUs) usually need information about the relationship between the device and the sensor, which depends on the mounting orientation. Based on this mounting orientation, it is possible to estimate the attitude of the device or vehicle [1], to implement strap-down-algorithms [2], and to analyze the direction of the motion and acceleration [3]. In some smartphones, for example, the relation between the two coordinate systems is obtained by placing the sensors in alignment with the device coordinate system [4]. In addition, the mounting orientation can be obtained offline based on high-quality accelerometer data and further information about the environment such as the slope of the road [1,5], or a specified horizontal surface [6,7]. Furthermore, the method in [8] uses measurement data of reference points for compensating the installation angles and attitude errors of an INS/GPS/LDS Target Tracker. In addition to the horizontal surface, the method in [6] uses the acceleration amplitude to estimate the yaw mounting angle. The approach in [9] evaluates the forward acceleration to identify the longitudinal axis of the vehicle. However, the accuracy of all these methods suffers considerably when using a consumer IMU sensor which is known to be associated with large errors.

**Figure 1:**
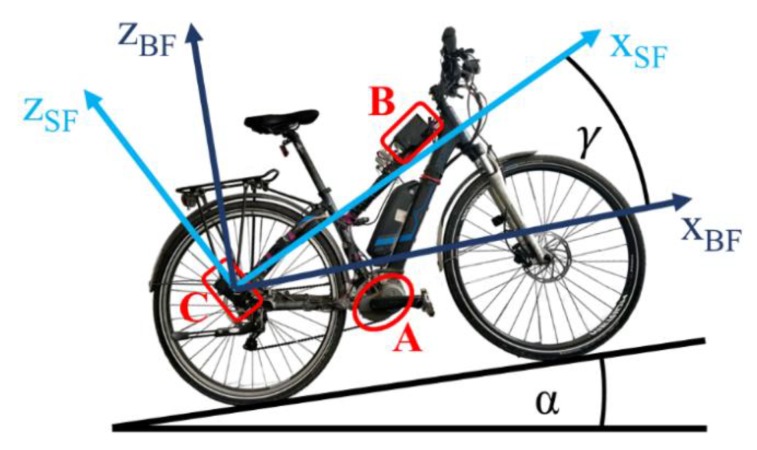
Experimental bike setup and representation of the sensor frame in the bike/vehicle frame [11] in a 2D system. (**A**) A mid-mounted motor; (**B**) a system on chip containing an atmospheric pressure sensor and a CAN (“controller area network”) interface; (**C**) a sensor box. The set up speedometer is a classic reed switch, located at the rear wheel.

For electric bicycles, there is a study on calibrating the orientation of flexion and extension axes of the lower extremities by using IMUs mounted on the bicyclist [10]. Existing approaches for calibrating the mounting orientation of IMUs placed on electronically power-assisted cycles (EPACs) are, in general, only methods for 2D-systems (see Figure 1). Such a 2D-correction is enough when the sensor is located in the motor unit of the EPAC. This is because the plane of the printed-circuit-board (PCB) of a mid-mounted motor always has a static parallel relation to the vertical bike frame plane to remain the catenary of the bike chain.

Nevertheless, the frame design provides one degree of freedom regarding the motor orientation, i.e., rotations around the out-of-plane axis. However, the motor mounting angle is very different in EPACs from different manufacturers, leading to different motor orientations. Thus, it is necessary to perform an online 2D-correction for transforming the x- and z-components of the IMU data from the sensor frame (SF) into the bike frame (BF).

Baumgärtner [12] analyzed the offset errors offline and calibrated the IMU orientation on an EPAC based on a priori knowledge about the road slope as in [5,6,7]. It was reported in [12] that the error of the estimated motor orientation required for the application of the method should be less than 3°. Based on the measured data during an uphill and downhill scenario on a road with a constant inclination, Ghislanzoni [13] proposed an approach to estimate the orientation of an accelerometer mounted on a bicycle. However, the results of this method are affected by the sensor biases. Furthermore, this method needs specific motions and thus the estimation cannot be performed in arbitrary cycling scenarios.

In addition, to the best of our knowledge, no method exists in the open literature for 3D-correction and calibration of an IMU which is mounted out of the bike frame and independent of sensor biases.

In this paper, we propose a novel auto-correction method to estimate the 2D- and 3D-orientation of IMUs on electric bicycles. The correction is based on the measured data of common bicycling motions such as accelerations and decelerations as well as roll and steer motions of the system. The bias-compensated mounting orientation is achieved with a speedometer. After transforming the accelerometer data into the vehicle coordinate system, we use a simple and robust method to estimate the sensor biases. In contrast to the method presented in [14], in our method, a bias-independent mounting orientation correction is carried out at first, and afterwards a sensor offset estimation and compensation is made. This leads to significant advantages regarding the accelerometer error analysis in the bike frame coordinate system. Therefore, an atmospheric pressure sensor is needed for the bias estimation. More importantly, the computation expense required for the estimation is very low so that it can be realized in real-time.

The remainder of this paper is organized as follows. In the next section, we present the challenge of the mounting orientation correction for consumer micro-electro-mechanical systems (MEMS) sensors with large offset errors. Section 3 introduces the proposed method, provides an analysis of dynamic bike motions, and proposes the solution for the 2D and 3D mounting correction. Section 4 presents experimental results to demonstrate the applicability of our method. Section 5 concludes the paper.

## 2. Challenge

**Figure 2:**
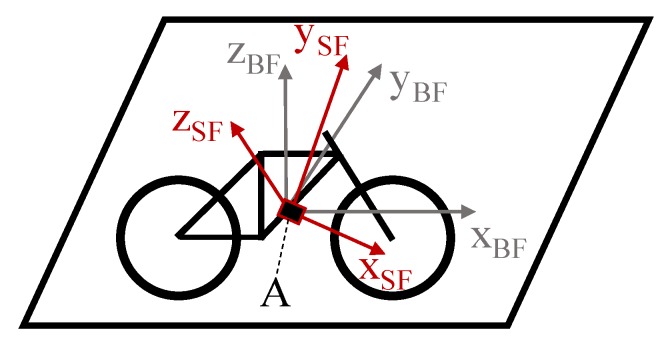
Depiction of the sensor frame system and the bike/vehicle frame system [11] (3D), where **A** represents the randomly orientated sensor.

In this study, we consider the estimation of the mounting orientation of IMU in an EPAC as shown in Figure 1 for a 2D and Figure 2 for a 3D description.

Based on the mounting orientation, the sensor data can be transformed from the sensor coordinate system (SF) into the bike coordinate system (BF).

If the slope *α* (in this paper *α* is negative for uphill sections and vice versa [10]) is known, the 2D mounting orientation *γ* can be determined by:

(1) $$\gamma =\mathrm{actan}\left(\frac{z_{SF,true}}{x_{SF,true}}\right)-\alpha$$

based on a measurement of the two accelerometer-axes *z_SF_* and *x_SF_* in the vertical bike frame plane, corresponding to the true/bias-free accelerations (*x_SF,true_*, *z_SF,true_*).

However, the biases (*x_SF,b_*, *z_SF,b_*) of the accelerometer sensor have a strong influence on the accuracy of γ using (1). This influence leads to a discrepancy of the true sensor values which have not been properly considered in previous studies [5,6,7,8,9,12]. Such offset errors exist commonly in the output of consumer MEMS sensors due to temperature effects, factory processes and aging [15]. Therefore, (1) is modified as:

(2) $$\gamma =\mathrm{actan}\left(\frac{z_{SF,m}-z_{SF,b}}{x_{SF,m}-x_{SF,b}}\right)-\alpha$$

Now, the mounting orientation *γ* cannot be determined by a single measurement of the acceleration due to the two more unknowns (*x_SF,b_*, *z_SF,b_*).

Although biases can be pre-estimated in the sensor frame using the state-of-the-art accelerometer models by solving an ellipsoid-fitting problem [16,17,18,19,20], these methods require several static measurement data with different sensor orientations. In addition, for the ellipsoid-fitting, only the gravity of 1 g must be kept during the measurement, which is impossible in bicycling. Some of these methods [17,18] use further sensors, e.g., a magnetometer. Moreover, most of these methods use an optimization approach like the Newton method [16,19], the quasi-Newton method [18] or the unscented Kalman filter [20] to solve the nonlinear ellipsoid-fitting problem. In [21] a linearization of the nonlinear system was used in fitting the model parameters. Renk et al. performed the fitting based on measured state trajectories during a sufficiently slow motion of a robot arm [18]. However, since these sufficiently slow moving states have to cover a large portion of the ellipsoid, such methods are not suitable for fitting parameters in accelerometer models placed on EPACs. In general, IMUs can be readily calibrated with a Kalman filter. Nevertheless, in some specific cases, as seen in [22], the occurring motion data is not sufficient for proper convergence of the filter states. Therefore, the existing filter approaches are not sufficient for the bicycle use case, too, as it has similarities to the task presented in [22]. Especially in cycling activities in flat areas, the roll and pitch angle of a bicycle exhibits only small variance regarding orientation changes, which will lead to poor performances when using the approaches in [16,17,18,19,20]

To evade the necessity of measurement data with different orientations, further sensors (e.g., high-grade GPS data) could be introduced to the Kalman Filter design as shown in [22].

Streit and Braeuer [23] estimated the bias of an accelerometer in one axis by observing the wheel speed. However, it requires the accelerometer to be pre-aligned with the bike frame, i.e., the mounting orientation has to be known, which means the IMU is already calibrated.

In addition to the bias, a consumer IMU also has scale and alignment errors. In general, for sensor orientation and attitude estimation, the misalignment and scaling errors of the accelerometer can be neglected [15,18], whereas the biases of consumer sensors are significant and can lead to an error of up to 10 [15].

Based on the above discussion, we conclude that an online correction of the 2D and 3D mounting orientation of a consumer IMU for electrical bicycles remains a challenge. To address this problem, both sensor biases and sensor orientation have to be estimated online.

In general, we can estimate the sensor biases first and the orientation second, or vice versa. In this study, the latter is considered, i.e., the sensor orientation estimation has to be independent of the sensor biases.

## 3. Method

Our aim in this study is to develop a method to calibrate the mounting orientation of a consumer IMU. The estimation should be based on the data from common bicycling motions. It means that the cyclist does not have to perform specific maneuvers for the estimation. For this purpose, we consider two common cycling motions: a deceleration and a lateral pendulum motion.

### 3.1. Deceleration

**Figure 3:**
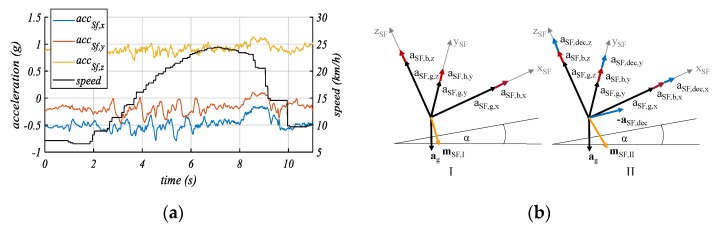
(**a**) Measured and filtered acceleration signals on an electric bicycle. The x- and z-axes are part of the vertical bike frame plane. (**b**) Schematic representation of acceleration components during phases without rider interactions (I) and during acceleration or deceleration of the bicycle (II) [24].

In principle, motions with acceleration and/or deceleration can be used for the mounting orientation estimation. During those motions, the direction points toward (by acceleration) or against (by deceleration) the driving direction of the bicycle. At first, we analyze the dynamic behavior with the help of typical profiles measured by an IMU on an EPAC. Figure 3a shows an acceleration phase from t = 2 s through t = 6 s and a deceleration phase from t = 8 s through t = 10 s. These phases can be recognized by the speed signal and the acceleration measurement in all three axes (*acc_Sf,x_*, *acc_Sf,y_*, *acc_Sf,z_*) caused by the mounting orientation of the IMU on the bicycle. During the acceleration phase, the oscillation appears because of the pedaling and steering control actions. More importantly, as shown in Figure 3a, the amplitudes of the deceleration are greater than those of acceleration, which is the typical case during cycling. Therefore, deceleration motions are used in our method.

Assuming that the bicycle does not change its orientation before and during the deceleration, we can estimate a bias-independent driving direction vector ***a****_SF,dec_*. The principle of the bias independence is presented in Figure 3b. It can be seen, by taking two deceleration measurements ***m****_SF_*_,*I*_ and ***m****_SF_*_,*II*_ (i.e., before and during braking) that we can isolate the deceleration components ($a_{\mathrm{SF},\mathrm{dec}}$) from the other vector components ($a_{\mathrm{SF},g},a_{\mathrm{SF},b})$ in the sensor frame. The two measurements represent the resulting acceleration ***m***_*SF*_. Since the orientation does not change, the bias vector ***a****_SF,b_* and the gravity vector ***a****_SF_*_,*g*_ remain constant. The deceleration vector ***a****_SF_*_,*dec*_ can be calculated as follows:

(3) $$a_{\mathrm{SF},\mathrm{dec}}=\left[\begin{array}{c} a_{SF,dec,x}\\ a_{SF,dec,y}\\ a_{SF,dec,z} \end{array}\right]=m_{\mathrm{SF},\mathrm{II}}-m_{\mathrm{SF},I}$$

where

(4) $$m_{\mathrm{SF},I}=a_{\mathrm{SF},g}+a_{\mathrm{SF},b}$$

(5) $$m_{\mathrm{SF},\mathrm{II}}=a_{\mathrm{SF},g}+a_{\mathrm{SF},b}+a_{\mathrm{dec}}$$

The constant orientation of the EPAC during the deceleration can be verified by the gyroscope of the IMU. This means that only braking situations without orientation change will be used.

It should be noted that the deceleration vector ***a****_SF_*_,*dec*_ is along the ***x****_BF_*-axis of the bike system (BF) [11]. Therefore, the vector ***a****_SF_*_,*dec*_ represents a link between the sensor and the bike coordinate system.

### 3.2. Lateral Dynamics

**Figure 4:**
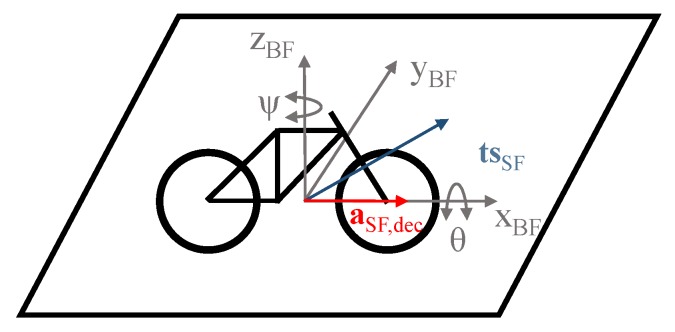
Roll motion *θ* and steer-control motion *ψ* in the vehicle system bike frame (BF). ***ts****_SF_* represents the combined motion vector. *a_SF,dec_* is the deceleration vector.

The inherent instability of the bicycle is mainly due to the unbalanced motion described by the lateral dynamics of cycling. Schwab and Meijaard [25] investigated this instability and presented an in-depth study on self-stabilizing mechanisms of the bicycle and the influencing factors of the cyclist. When the bicycle is tending to fall to one side, the cyclist has to steer in the direction of the undesired fall to avoid a crash. The related centrifugal acceleration sets the bike upright again. This is in fact the behavior of an inverted pendulum, leading to the oscillation shown in Figure 3a. The lateral dynamics of cycling can be described with two separate motions. The first one is the sideway rolling or tipping, leading to a rotation *θ* around the ***x_BF_***-axis. The second one is the steering motion to keep the balance, which ends up with a rotation *ψ* around the ***z_BF_***-axis. Thus, the combination of both rotations leads to a rotation around a vector ***ts****_SF_* (tip-steer) which is part of the vertical bike frame plane *x**_BF_z_BF_***, representing another link to the BF. Figure 4 illustrates this relation and the two rotations, where the vector ***ts****_SF_* will be estimated by analyzing the gyroscope data of the IMU in the frequency space.

**Figure 5:**
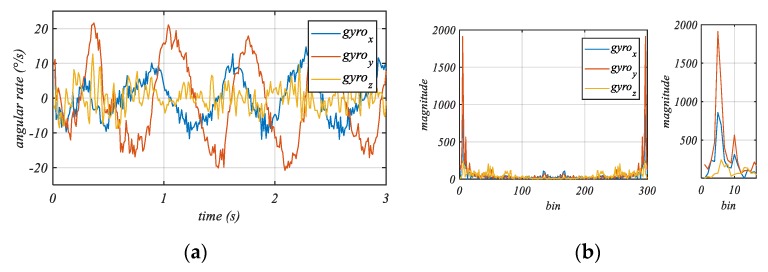
(**a**) Gyroscope data during pedaling series representing the lateral effects of the tip-steer motion. (**b**) Frequency spectrum of gyroscope data during pedaling and a more detailed plot of the lower part of the frequency spectrum.

Typical profiles of gyroscope data recorded during a pedaling phase are plotted in Figure 5a where a predominant oscillation in all three axes can be observed. The phase shift of the profiles shows the temporal relation between the tip motion and the steer motion, reflecting the delayed reaction time of the cyclist. In Figure 5b, the frequency spectrum of the data shown in Figure 5a is plotted, which is computed with a fast Fourier transformation (FFT) with a sampling rate *F_s_* 100 Hz using 300 samples. It can be seen that one peak (i.e., the maximum magnitude) is at the lower end of the spectrum due to the tip-steer motion (***ts****_SF_*).

Kooijman et al. [26] pointed out that during pedaling the inverted pendulum motion has a frequency equal to the pedaling frequency (cadence). This correlation between the cadence and the maximum magnitude in the frequency spectrum can be observed in the data shown in Figure 5. As a result, evaluating only one single pedaling frequency of the gyroscope data is enough for our purpose and, thus, the computation expense can be significantly reduced. This is made by a convolution or a single multiplication of the current 3 × n gyroscope dataset ω and a cosine function describing the current cyclist cadence F_Cad_. The rotational rate matrix ω has three columns representing the three rotational rate axes of the gyroscope and *n* rows corresponding to the number of recorded data points. To calculate the components of the tip-steer motion vector, we need the integration of the absolute convolved or multiplied function values. For the convenience of computation, we calculate the sum of the products $\vartheta_{F_{Cad}}$ of the discrete signals, which is the unsigned vector ***ts****_SF_*:

(6) $$\vartheta_{F_{Cad}}=\left|ts_{SF}\right|=\overset{n}{\underset{k=0}{\sum }}\left|\omega_{k}*\cos\left(\frac{2*\pi *k*F_{Cad}}{F_{s}}\right)\right|$$

This computation can be interpreted as a product of a measured time series of the sensor data and a specific cosine function with the frequency of the current cyclist cadence.

The summation of the products returns the unsigned tip-steer vector $\vartheta_{F_{Cad}}$. This procedure can be derived from a discrete Fourier transformation without an imaginary component and an evaluation of only one defined frequency [27].

Due to the unsigned magnitudes of the frequency spectrum and the necessity of a signed tip-steer vector, the signs of $\vartheta_{F_{Cad}}$ in (6) have to be determined separately. In total, there are eight possible sign combinations for a vector with three components. Two combinations are always mirrored vectors; these vectors are the same except for their opposite direction. Thus, considering the fact that four sign variations are enough, this leads to a further reduction of the computation time. Each sign variation of ***ts****_SF_* is multiplied by ω to calculate the four one-dimensional-rotational rate signals. The signal with the maximal amplitude represents the true tip-steer motion and exhibits the correct sign variation.

It should be noted that the estimation of the tip-steer vector ***ts****_SF_* is independent of the gyroscope biases, too, because the method of frequency analysis evaluates only the content of the non-zero frequency data of the gyroscope.

### 3.3. From Sensor Frame to Bike Frame

#### 3.3.1. Two-Dimensional (2D) Mounting Orientation Estimation

Here, we present a method for a 2D-auto-correction in which only the deceleration vector ***a****_SF,dec_* is used. This vector is the input for the rotation matrix R2DSfBf used for transforming the *x_SF_*- and *z_SF_*-axes of the sensor frame into the driving (***x_BF_***) and vertical (***z_BF_***) axes of the BF (see Figure 1). Therefore, the rotation angle *γ* between the two coordinate systems is independent of the slope *α* due to the compensation of the gravity (see (3)–(5)) and thus can be calculated by

(7) $$\gamma =\mathrm{actan}\;\left(\frac{a_{\mathrm{SF},\mathrm{dec},z}}{a_{\mathrm{SF},\mathrm{dec},x}}\right)$$

Since the *y*-axis of the sensor frame remains the *y*-axis of the bike frame for the 2D case, we use the following standard rotation matrix

(8) R2DSfBf=[sin (γ)0−cos (γ)010cos (γ)0sin (γ)]

#### 3.3.2. Three-Dimensional (3D) Mounting Orientation Estimation

For the 3D-auto-correction, both the deceleration and the lateral pendulum motions discussed above are required. By calculating the cross product of the vector ***ts****_SF_* and the deceleration ***a****_SF,dec_*, and since these two motions occur in the sensor frame, we can estimate the lateral axis ***y****_BF_* as a relation to the bike frame (see Figure 4):

(9) $$y_{BF}=\frac{ts_{SF}\times a_{SF,dec}}{\left|ts_{SF}\times a_{SF,dec}\right|}$$

The driving direction axis ***x**_BF_* represents the sign-inverted and normed deceleration vector ***a**_SF,dec_*, i.e., $x_{BF}=\frac{-a_{SF,dec}}{\left|a_{SF,dec}\right|}$ The vertical axis ***z_BF_*** is calculated as the cross product of ***x****_BF_* and ***y****_BF_*:

(10) $$z_{BF}=x_{BF}\times y_{BF}$$

As a result, the rotation matrix R3DSfBf transforms the sensor data from the sensor frame to the bike frame:

(11) R3DSfBf=[xBFTyBFTzBFT]

The acceleration vector $m_{acc,BF}$ in the BF is the product of the rotation matrix RSfBf and the acceleration vector $m_{acc,SF}$, which can be expressed for the 2D and 3D rotation matrices as follows

(12) macc,BF=RSfBf∗macc,SF

### 3.4. Bias Estimation in the Bike Frame

As mentioned above, due to the data inaccuracy of a consumer IMU, it is necessary to estimate the sensor biases. The calibration of the gyroscope biases is usually undertaken during phases in which the device is in a “constant position” [28]. However, the accelerometer biases cannot be determined with the data of a static state [16]. Thus, we need more cycling scenarios or events together with a standard low-cost bicycle speedometer and an atmospheric pressure sensor to determine the acceleration biases.

The current road slope can be evaluated by integrating the speedometer data [29,30] to obtain the distance *ds* and estimating the change of the altitude *dh* based on the barometer data [31]. Then, we can calculate the slope:

(13) $$\alpha =\arcsin\left(\frac{dh}{ds}\right)$$

However, the estimation of *α* in this way is delayed due to the necessary cycling distance *ds* as well as a low-pass filter to remove the noise of the pressure signal. Considering these aspects, we determine the biases $b_{acc,BF,x}$ in the driving direction and $b_{acc,BF,z}$ in the vertical direction by comparing the measured acceleration $m_{acc,BF,LP}$ with a gravitational component at the slope *α* during constant speed events. The subtraction of the measured accelerations from the gravitational components represents the acceleration biases $b_{acc,BF,x}$ and $b_{acc,BF,z}$.

The lateral acceleration bias *b_acc,BF,y_* can be estimated based on the consideration that, during pedaling events, the bicycle is, in general, upright. Consequently, all measured and low-pass-filtered acceleration $m_{acc,BF,y,LP}$ corresponds to the lateral bias $b_{acc,BF,y}$. Therefore, the acceleration biases $b_{acc,BF}$ in the three axes are:

(14) $$\left[\begin{array}{c} b_{acc,BF,x}\\ b_{acc,BF,y}\\ b_{acc,BF,z} \end{array}\right]=\left[\begin{array}{c} 1g*\sin\left(\alpha \right)-m_{acc,BF,x,LP}\\ m_{acc,BF,y,LP}\\ 1g*\cos\left(\alpha \right)-m_{acc,BF,z,LP} \end{array}\right]$$

### 3.5. Implementation of the Proposed Approach

**Figure 6:**
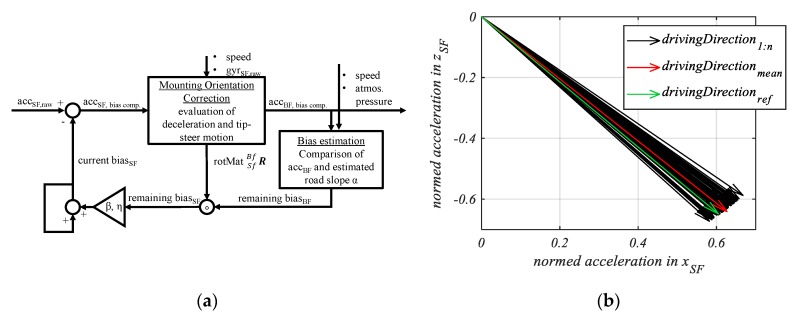
(**a**) Block diagram of the estimation algorithm. (**b**) [24] Compensated driving direction vectors, based on deceleration vectors (*n* = 55) in the sensor frame, reference driving direction is measured offline by an offset stable inertial measurement unit (IMU) [32], rigid fork setup.

Figure 6a shows the implementation of the overall method. The input data are the measured acceleration, rotational rate, speed, and atmospheric pressure.

In the forward path, the mounting orientation is estimated and the acceleration data transformed into the BF. The feedback path presents the event-based bias estimation. These events are cycling scenarios with constant speeds.

The residual of the biases $b_{acc,BF}$ is estimated, transformed into the sensor frame and sent back to the forward path as a bias compensation. This feedback loop is similar to a proportional controller (i.e., the last bias combined with the new estimated remaining bias, existing in the current data), where *β, η* are the proportional parameters to be tuned. These two parameters are necessary for different handling of the lateral bias *b_acc,SF,y_* and the other two biases (*b_acc,SF,x_*, *b_acc,SF,z_*).

Two MATLAB/Simulink models are implemented to realize our 2D- as well as 3D-auto-correction method, respectively.

## 4. Results and Discussion

### 4.1. Hardware Setup

The EPAC used for the experiment is a standard electrified trekking bicycle (see Figure 1). Two test series were carried out: one with a rigid fork and one with an unlocked suspension fork (suspension travel < 70 mm). Using these two configurations, we want to check the influence of the suspension elements on the deceleration vector. The consumer IMU used is the BMI160 [14] set up in its normal mode at a range of ± 16 g and 2000/s. In addition, three automotive grade sensors were used as a reference for verifying the results. These reference sensors have a high offset stability over temperature and lifetime [32]. They were aligned manually with the consumer IMU on the EPAC frame to evaluate the estimated acceleration biases by using the novel approach. The reference mounting angle(s) were measured offline on flat ground.

The standard bicycle speedometer used is a classic reed switch, located at the rear wheel. It generates one impulse per rotation. Every time a new impulse is detected, a speed update is given.

### 4.2. Data Acquisition

The deceleration phases were detected by the speedometer signal. Only measured data of braking motions without orientation changes were used for the estimation and verified by the measured gyroscope data. For the 3D-auto-correction, the gyroscope data were saved during pedaling to estimate the tip-steer vector. The data was sampled and the estimation performed at 100 Hz. Since all sensor signals are sampled from the same system-on-chip with the same data rates and corresponding timestamps, synchronization is achieved.

### 4.3. Experimental Results

#### 4.3.1. Two-Dimensional (2D) Mounting Orientation Estimation and Deceleration Vector Estimation

At the beginning, the EPAC with a rigid fork was used to test both the functionality of our 2D method and the estimation of the deceleration vector. The test data consisted of 55 detected braking phases, where the IMU was aligned with the vertical bike frame plane *x_BF_z_BF_*. Decelerations were performed on different road slopes, in the range of 0.7 m/s^2^–2.6 m/s^2^.

Thus, the recorded braking phases were in the range of common cycling decelerations which were lower than the critical decelerations with 6 m/s^2^ [33].

Figure 6b shows the driving direction vectors based on the measured decelerations. The reference mounting angle *γ_ref_* was determined to be 44.9. The mean deceleration of the measured data led to a mounting angle *γ_mean_* of 45.6. As a result, the estimation error was less than 1.

In the next step, we tested the setup with the unlocked suspension fork. In this case, we expected that a greater error due to the dipping of the suspension fork would occur. From a test drive, 46 decelerations were recorded and evaluated. The reference mounting angle *γ_ref_* was determined to be 48.1. The mean deceleration of the measured data led to a corresponding mounting angle *γ_mean_* of 46.5, i.e., the estimation error was less than 2. In addition, the result shows that the online estimated mounting angle was smaller than the reference angle due to the dipping motion.

#### 4.3.2. Three-Dimensional (3D)-Auto-Correction

For the verification of the 3D-auto-correction method, an IMU was mounted at the lower part of the luggage rack, close to the rear wheel axle. A cycling test with 15 min was recorded, in which 11 braking situations were detected. The reason for this relatively high number of braking events in such a short cycling time is that the first 7 braking situations were deliberately executed to allow the correction method to converge faster to the mounting orientation and bias estimation.

**Figure 7:**
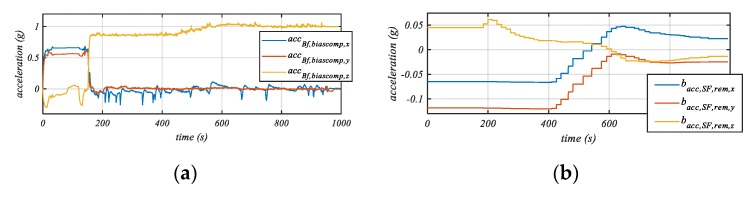
(**a**) Acceleration data in the bike frame. A first correction is done after 160 s. The period before this time represents the uncorrected data in the sensor frame, as the initial rotation matrix is the identity matrix. Consecutively the bias estimation and compensations starts. (**b**) Acceleration biases in the sensor frame, determined by comparing with automotive-grade bias a stable reference IMU [32].

Figure 7a shows the measured acceleration profiles in the bike frame. It can be seen that, in the beginning phase, the signals reflect the sensor frame data. After 160 s, necessary cycling motions were detected and a first estimation of the mounting orientation was made. The estimation was conducted continuously, i.e., online. It can be seen that the *z*-axis shows the gravity, and meanwhile, in the x- and *y*-axes there are almost no accelerations except for the acceleration and deceleration motions of the bike and centrifugal accelerations in turning situations. At 400 s, the main bias estimation began. This can be observed in the *z* axis of the bike frame coordinate system (Figure 7a). In Figure 7b, the biases of the acceleration signals remaining in the sensor frame can be seen. As expected, the estimated biases show the same chronological behavior as the biases shown in Figure 7a. Furthermore, it can be perceived that each bias parameter in the bike frame coordinate system has an influence on all biases in the sensor frame coordinate system, due to the coordinate transformation.

Based on the estimated rotation matrix, the errors of the mounting orientation were determined as angular errors, showing the difference of the reference orientation from the online estimated orientation. In the test case, the angular errors were below 2 in all axes.

It can be seen from Figure 7a that the maximum measured bias was −0.12 g in the *y_SF_*-axis. In comparison, the maximum bias of the consumer sensor used is reported in [15] as 0.15 g. In addition, the accuracy of the bias estimation was compared with the data from the offset-stable reference sensors [29] aligned with the consumer IMU. After 15 min of cycling and detecting several necessary motions, the maximum bias was about 0.02 g, leading to a relative reduction of 80%. With further tests evaluated, it is shown that the average value of the deceleration vector and the tip-steer vector were further improved regarding the mounting orientation correction.

The robustness of our method was evaluated by a study with more cyclists and the result is shown in Table 1. For all tests, the maximum orientation estimation error was below 2.5 and more than 80% of the acceleration biases were eliminated on average.

Furthermore, we analyzed the performance of our method for bike motions in daily cycling; 20 tests with different cyclists and different electric bicycles were carried out. However, these bicycles were not equipped with the reference sensors. Here, we wanted to investigate the performance of our method in more cycling scenarios with various cyclists and different bicycles. The cyclists were not instructed to perform specific motions before the tests.

**Figure 8:**
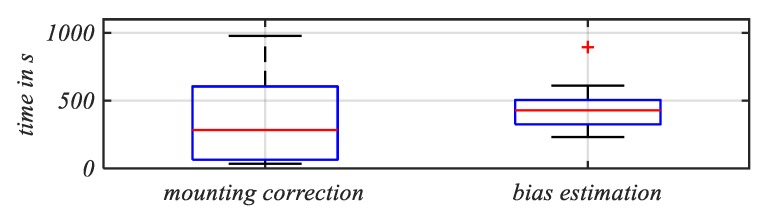
Box plot, demonstrating the results of the second multi-cyclist analysis, regarding the median time until all necessary bike motions are detected and the downstream bias estimation converged.

The durations of the tests were from 7 to 86 min. The results are presented in Table 2 as well as in Figure 8. It can be seen that only two out of the 20 cyclists (No. 11 and 18) did not perform braking motions (i.e., without bike orientation change). The other cyclists exhibited motions necessary for the mounting orientation correction. The period for activating the orientation estimation was highly different (see Figure 8), due to different riding behaviors of the cyclists. However, the time taken for the convergence of the bias estimation did not vary strongly, since the necessary events (i.e., pedaling and constant speed) occurred much more often than the braking events for the mounting estimation.

## 5. Conclusions

In this paper, we proposed a novel approach for estimating 2D orientation of an IMU located in the vertical bike frame plane based on common cycling motions. Our method is independent of acceleration biases, thus allowing the use of low-cost consumer IMUs. Our experimental results show high estimation accuracy, i.e., the standard error for EPACs without suspension components was less than 1 in the 2D case.

The extension of the method to 3D-auto-correction based on tip-steer-motions exhibits satisfactory results as well. It is shown from the experimental results that the error between the true coordinate system and the estimated bike frame system was less than 2.5. The bias estimation using the data of an atmospheric pressure sensor was able to reduce the acceleration biases of a consumer IMU by approximately 80%. Tests including multiple cyclists with different bike setups demonstrated the applicability of our method in daily cycling of different cyclists. As a result, our method leads to a significant benefit for auto-correction of 3D mounting orientation and sensor bias compensation of IMUs in electric bicycles used in daily scenarios. The high accuracy achieved by our estimation method allows many applications like drive-off detection, brake detection and attitude estimation with a calibrated consumer IMU.

## 6. Patents

DE102015115282A1 resulted from this work.

## Figures and Tables

**Table 1:** Experimental multi-cyclist analysis regarding estimation accuracy.

Cyclist	Riding Time in S	Amount of Evaluable Braking/Tip Steer Situations	Max. Mounting Orientation Error in	Relative Bias Reduction/Max. Remaining Bias
1	3040	4/562	2.4	79.6%/0.019 g
2	1980	11/287	0.6	89.7%/0.009 g
3	2569	9/219	1.6	79.4%/0.022 g
4	2040	10/220	2.0	86.7%/0.014 g
5	882	4/59	1.3	78.0%/0.022 g
6	2667	11/251	1.2	78.7%/0.026 g
7	2756	13/398	1.2	81.0%/0.019 g
8	2861	9/443	2.3	66.0%/0.034 g

**Table 2:** Experimental multi-cyclist analysis regarding convergence of 3D sensor mounting correction and bias estimation.

Cyclist	Time in S, Till Bike Motions Detected	Time in S, between Motion Detection and Convergence of Bias Estimation	Convergence of Bias Estimation	Total Length of Testride in S
1	268	557	converged	2940
2	40	325	converged	953
3	396	256	converged	5213
4	277	426	converged	1155
5	486	154	converged	2387
6	282	−	no final Convergence	462
7	34	274	converged	823
8	286	335	converged	1365
9	605	895	converged	2257
10	65	−	no final Convergence	538
11	noBrakeDetected	−	no Convergence	1897
12	34	446	converged	2292
13	132	433	converged	1891
14	54	506	converged	1496
15	810	−	no final Convergence	853
16	776	478	converged	4278
17	979	231	converged	1318
18	noBrakeDetected	−	no Convergence	670
19	654	611	converged	3061
20	323	331	converged	1522
